# Reconstructing the ubiquitin network - cross-talk with other systems and identification of novel functions

**DOI:** 10.1186/gb-2009-10-3-r33

**Published:** 2009-03-30

**Authors:** Thiago M Venancio, S Balaji, Lakshminarayan M Iyer, L Aravind

**Affiliations:** 1 National Center for Biotechnology Information, National Library of Medicine, National Institutes of Health, Bethesda, Maryland 20894, USA

## Abstract

**Background:**

The ubiquitin system (Ub-system) can be defined as the ensemble of components including Ub/ubiquitin-like proteins, their conjugation and deconjugation apparatus, binding partners and the proteasomal system. While several studies have concentrated on structure-function relationships and evolution of individual components of the Ub-system, a study of the system as a whole is largely lacking.

**Results:**

Using numerous genome-scale datasets, we assemble for the first time a comprehensive reconstruction of the budding yeast Ub-system, revealing static and dynamic properties. We devised two novel representations, the rank plot to understand the functional diversification of different components and the clique-specific point-wise mutual-information network to identify significant interactions in the Ub-system.

**Conclusions:**

Using these representations, evidence is provided for the functional diversification of components such as SUMO-dependent Ub-ligases. We also identify novel components of SCF (Skp1-cullin-F-box)-dependent complexes, receptors in the ERAD (endoplasmic reticulum associated degradation) system and a key role for Sus1 in coordinating multiple Ub-related processes in chromatin dynamics. We present evidence for a major impact of the Ub-system on large parts of the proteome via its interaction with the transcription regulatory network. Furthermore, the dynamics of the Ub-network suggests that Ub and SUMO modifications might function cooperatively with transcription control in regulating cell-cycle-stage-specific complexes and in reinforcing periodicities in gene expression. Combined with evolutionary information, the structure of this network helps in understanding the lineage-specific expansion of SCF complexes with a potential role in pathogen response and the origin of the ERAD and ESCRT systems.

## Abstract 2

A computational model of the yeast Ubiquitin system highlights interesting biological features including functional interactions between components and interplay with other regulatory mechanisms.

## Background

Post-translational modification of lysine, serine, threonine, tyrosine, aspartate, arginine and proline residues in proteins are widely observed and are of paramount importance in the regulation of several cellular processes. These modifications range from linkages of low molecular weight moieties, such as hydroxyl, phosphate, acetyl or methyl groups, to entire polypeptides. Covalent modification by protein tags, which involves linkage of polypeptides belonging to the ubiquitin (Ub)-like superfamily, to target lysine (rarely cysteines or amino groups of proteins) is best understood in eukaryotes. In addition to Ub, these protein modifiers include a variety of other Ub-like polypeptides (Ubls), such as SUMO, Nedd8 and Urm1 [1]. Modification of a target by an Ub or Ubl can take many different forms and can have many diverse consequences [1]. For example, polyubiquitination via lysine 48 (K48), as well as neddylation and urmylation can have destabilizing effects on the target by recruiting it for proteasomal degradation. In contrast, polyubiquitination via K63, monoubiquitination and sumoylation result in altered properties and interactions of the localized protein, thus having a primarily regulatory impact [2]. In particular, sumoylation has been implicated in the regulation of several functions, such as nucleocytoplasmic transport, cell cycle progression, nuclear pore complex-associated interactions, DNA repair and replication and mRNA quality control (reviewed in [3-5]). Other modifications, like that by Apg12, mediate specific biological processes such as autophagy [6].

Ub/Ubl modifications are achieved by an elaborate system involving several enzymes and regulatory components that are intimately linked to the proteasome [7]. Firstly, Ub and the Ubls might be processed from a longer precursor protein by proteases to expose the carboxyl group of the carboxy-terminal glycine. The conjugation process itself involves a three enzyme cascade, namely E1, E2 and E3. Of these, the E1 enzyme usually catalyzes two reactions - ATP-dependent adenylation of the carboxylate followed by thiocarboxylate formation with an internal cysteine in the E1. This is followed by a trans-thiolation reaction that transfers Ub/Ubl to the active cysteine of the E2 enzyme. E2s then directly transfer the Ub/Ubl to the target lysine, often aided by the E3 ligase [2,7,8]. The primary component of E3 ligases is the RING finger domain or a related treble-clef fold domain, such as the A20 finger [2,9]. E3 ligases also often contain other subunits such as F-box domain proteins, cullins and POZ domain proteins (for example, Skp1 in yeast). Alternatively, Ub/Ubls can be transferred by a further trans-thiolation reaction to HECT E3 ligases, which then transfer the Ub/Ubl to substrates. In many cases multiple rounds of ubiquitination of the initial oligo-Ub adduct are catalyzed by a specialized E3 that contains a derived version of the RING finger called the U-box, resulting in poly-Ub adducts [9,10]. Interaction of Ub chains on target proteins with the proteasome is also an intricate process involving specialized Ub/Ubl receptors and adaptors, which recognize Ub via domains such as the UBA, Little Finger, UIM, and PH domains [11]. Further Ub/Ubls attached to targets are recycled at the proteasome by de-ubiquitinating peptidases (DUBs) containing the JAB metallopeptidase domain. Other DUBs, belonging to diverse superfamilies of peptidases, usually have a regulatory role in removing Ub/Ubls from various targets [12]. Typically, DUBs are also the same proteases involved in releasing Ub/Ubls from their polyprotein precursors and show a relationship to viral proteases involved in viral polyprotein processing [12-14]. In addition to these core components, several other components are involved either as auxiliary, specificity-related subunits, or as scaffolds or as chaperones.

We term this total system comprising core components directly involved in Ub conjugation, removal/recycling and their accessory partners as the Ub-system. While earlier work by others and our group has investigated the provenance and evolution of individual components of this Ub-system [8,13,14], few studies have sought to acquire a holistic picture of the entire system. This has recently become possible, at least in a well-studied model eukaryote like *Saccharomyces cerevisiae*, as a result of the coming together of numerous technical and informational advances. First, genome-scale biochemical and proteomics studies have produced enormous amounts of data of diverse types, such as on protein-protein interaction [15-18], targets of ubiquitination [19-23] and sumoylation [24-28], and protein stability [29], abundance [30,31] and subcellular localization [32]. Second, several specific studies have determined interactions of the E3 ligase Rsp5 [33] and the proteasome subunit Rpn10 [20,21]. Third, case-by-case functional studies, coupled with highly sensitive sequence profile comparison methods, have enabled a comprehensive identification of Ub-system proteins with a high degree of confidence. We exploited the above advances to comprehensively identify Ub-system components in yeast and then assemble all their known physical, genetic and biochemical interactions between themselves and with the rest of the proteome. Graphs or networks have become the standard representation of such datasets in studies adopting a 'systems' approach. Such representations have enabled application of graph theoretic methods to extract previously concealed information regarding the system as a whole. They have been successful in analyzing other systems, such as the transcriptional regulatory network and protein interaction networks [34-36]. We accordingly represent our reconstruction of the Ub-system as a network, henceforth called U-net (for ubiquitin network). By analyzing the U-net, we were able to uncover several interesting biological features of the Ub-system, both in terms of previously unclear functional interactions of its components, as well as its interplay with other regulatory mechanisms, such as transcriptional regulation. As a result, we were also able to obtain the first objective quantitative measure of the impact of the Ub-system on cellular functions.

## Results and discussion

### Analysis of the ubiquitin system as a network

#### Assembly of the *Saccharomyces cerevisiae *U-net

**Figure 1:**
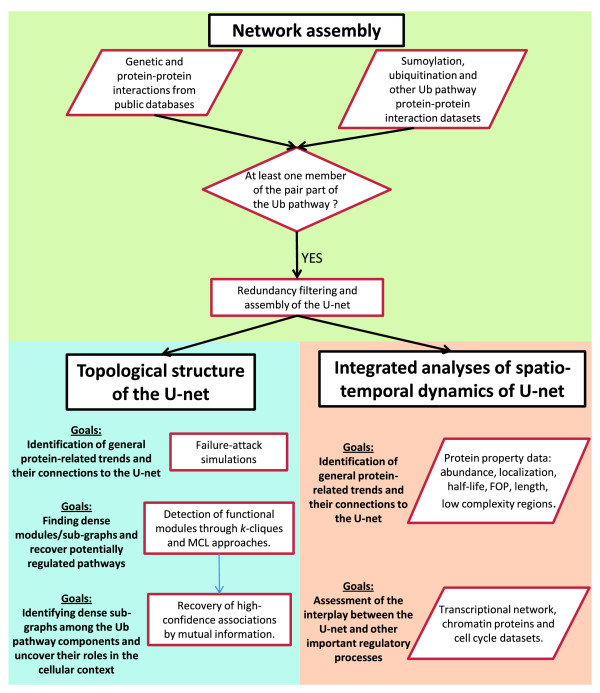
Flowchart for reconstruction of the U-net and its analysis. The flowchart describes the construction of the network, followed by analyses of topological structure and integration of different datasets for biological inference. FOP: Frequency of optimal codons.****

To assemble the *S. cerevisiae *U-net, we gathered all identified components of the Ub-system by means of literature searches and classified them according to the conserved protein domains present in them. Sensitive sequence profile analyses of each of the protein domain families were performed to identify all possible paralogs in the genome. We then surveyed all newly identified proteins based on domain architectures, catalytic active sites in the case of enzymes and binding pockets in other cases (when known), presence of functionally non-diagnostic and promiscuously fused protein domains and available literature. Having thus filtered out potentially irrelevant proteins, we arrived at a high confidence list of components of the *S. cerevisiae *Ub-system that is more comprehensive than any previously published list of this type (Figure 1; File S1 and Table S1 in Additional data file 1). In the process we made several new observations, including identifications of previously unknown representatives of certain domains. For example, we discovered that Ynl155w contains a novel SUMO-like Ubl domain and that Def1, which mediates ubiquitination and proteolysis of the RNA polymerase present in an elongation complex [37], contains an amino-terminal CUE domain that is likely to be critical for its interaction with Ub.

Using this list of components as the basis, we assembled the U-net by integrating an enormous volume of genetic and protein-protein interaction data obtained from public databases and specific case-studies in the literature on the Ub-system (see Materials and methods for details). By comparing individual protein-protein and genetic interaction datasets with lists of Ub/Ubl modified targets, we were able to show that the majority of these post-translational modifications are likely to be transient (that is, rapid protein degradation or Ubl removal) or condition-specific. Hence, they are almost completely missed by the high-throughput protein-protein interaction datasets. To address this lacuna, we incorporated both large-scale proteomic and individual case-by-case studies of Ub/Ubl modifications of proteins to reconstruct a more complete picture of the U-net (Figure 1). As these data are generated from proteins purified directly from cells followed by detection of modifications by mass-spectrometry, they are less likely to be affected by biases of *in vitro *modification assays where targets are specifically chosen. However, it should be mentioned that our reconstruction of the U-net is beset by the issue of a lack of temporal or condition-specific resolution, because most interactions were obtained under standard growth conditions. Further, one also needs to bear in mind the caveat of incompleteness of the available interactome and inherent limitations of different biochemical techniques. Questions have been raised about the quality of different interactome-determination techniques. However, a recent study provides evidence that the two main techniques used to detect protein-protein interactions, namely yeast two-hybrid and affinity-purification-coupled mass spectrometry are of high quality and of complementary natures [36]. Hence, we decided to use all available data, rather than filtering the data and lending greater weight to a particular technique (Figure 1).

#### Basic structure and properties of the U-net

**Figure 2:**
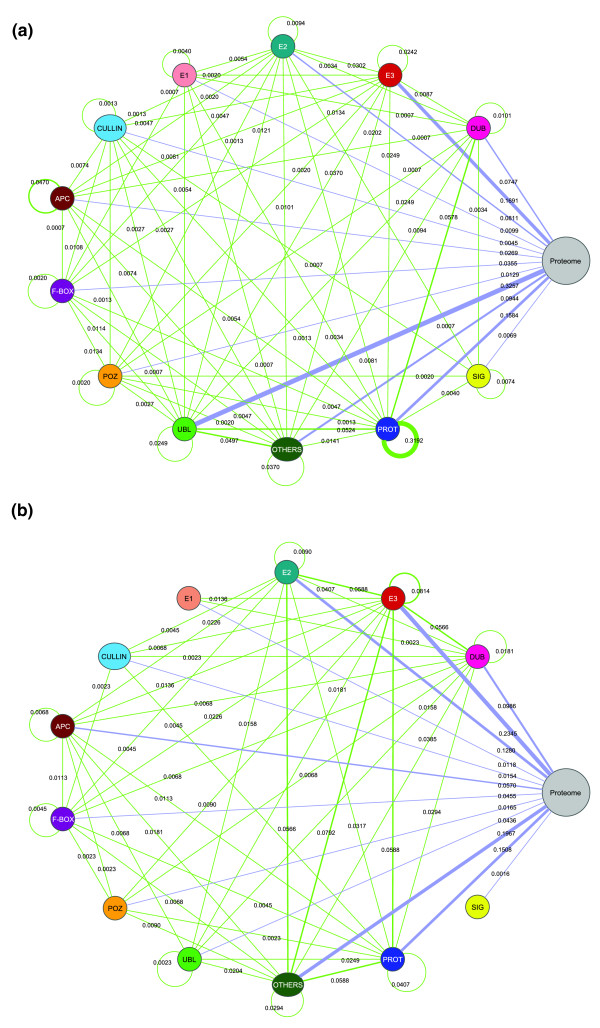
U-net classes and their interactions. The graph represents the Ub pathway wherein individual nodes have been collapsed into their respective general protein classes. The different contributions of **(a) **protein-protein and **(b) **genetic interactions that contribute to the overall U-net are shown separately. The proteome represents the rest of the proteome (that is, minus the Ub-system). The U-net-spec connections are shown in green while those to the proteome are shown in mauve. The intra-proteasomal protein-protein interactions are seen to stand out in graph. The figure also implies that only a fraction of the modifications are reversed by the DUBs.

The thus obtained U-net is an undirected graph, composed of 3,954 proteins (nodes) and 15,487 interactions (edges) representing genetic and protein-protein interactions of both covalent and non-covalent types (Figure 2; File S1 in Additional data file 1). Within the U-net a subnetwork can be identified, which is composed of all interactions between Ub-system components themselves, hereafter referred to as U-net-spec (for Ub specific network; Table S1 in Additional data file 1). In the U-net-spec the largest contribution is from protein-protein interactions of proteasome components (approximately 31.9% of U-net-spec interactions), which is reflective of the proteasome being a tightly interacting large protein complex (Figure 2a). In terms of connections to the rest of the proteome, there is a progression of increasing number of interactions in the order E1-E2-E3-Ub/Ubls (Figure 2a, b). This order is consistent with the observed biochemistry of the Ub-system, where there is increasing target specificity along the E1-E2-E3 enzyme cascade, with several E3s adding Ub/Ubls to more than one substrate [7]. As expected, Ub and SUMO are the two primary hubs (that is, highly connected nodes; Table S1 in Additional data file 1) in the network as they connect to a significant part of the proteome through direct covalent linkage. Other major hubs are the E2s Ubc7 and Rad6 (601 and 300 interactions, respectively), the E3 Rsp5 (376 connections) and the non-ATPase proteasomal subunit Rpn10 (432 connections) (all the information on connections and annotations are available in Table S1 in Additional data file 1).

**Figure 3:**
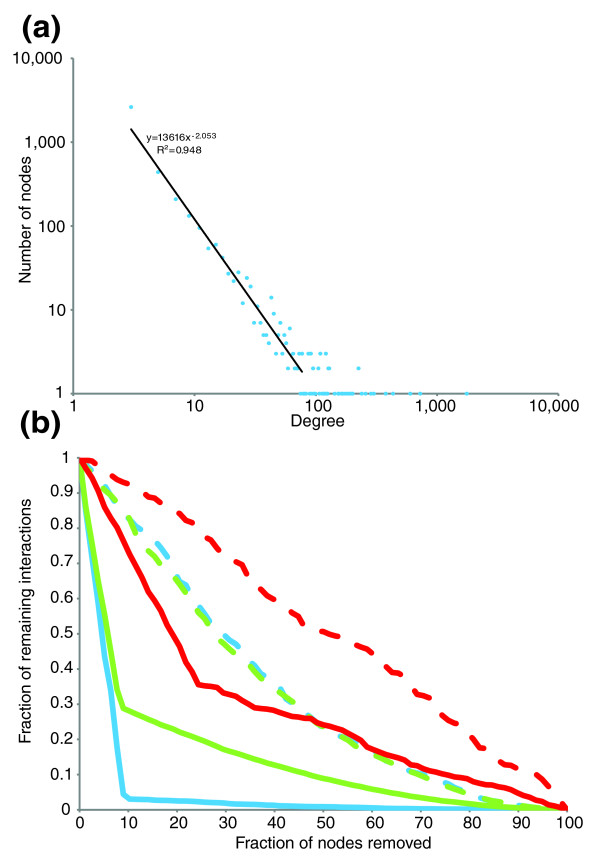
U-net (a) degree distribution and (b) tolerance to attack and failure. The U-net degree distribution is well approximated by a power-law equation: y = 13616x^-2.053 ^and R^2 ^= 0.948. The power-law distribution is common to several biological networks and is frequently associated with the scale-free structure and tolerance to failure [110].

Though the U-net, like most common biological networks [38], shows a degree distribution that is best approximated by a power-law (y = 13,616x^-2.053 ^and R^2 ^= 0.948; Figure 3a), it has several unique features. For example, the U-net is strikingly more susceptible to preferential disruption of its hubs (attack) in comparison to the transcriptional regulatory network (T-net) and the protein-protein network (P-net) - less than 5% of the total interactions remain upon simulated removal of a mere approximately 9% of nodes selected randomly amongst the hubs (Figure 3b). In terms of susceptibility to failure - that is, random removal of nodes - the U-net followed similar trends as the P-net, but the T-net was much more robust to failure than either of the former networks [34,39] (Figure 3b). We then surveyed the distribution of essential genes [40] and genes required for normal growth under environmental stress conditions (environmental stress response genes) [41] in the U-net. Hubs of the U-net were not enriched in any of these genes, suggesting that the high attack susceptibility of the U-net is unlikely to cripple the cell completely. In contrast, the U-net in general is enriched in essential genes relative to the entire proteome (the U-net contains about 78.6% of all essential genes, *P *≈ 4.914 × 10^-11 ^by Fisher exact test (FET); *P *≈ 4.711 × 10^-5 ^for environmental stress response genes by FET). This observation underscores the nature of the Ub-system as a predominantly regulatory system that operates on several essential functions, as opposed to being a basic 'house-keeping' system.

**Figure 4:**
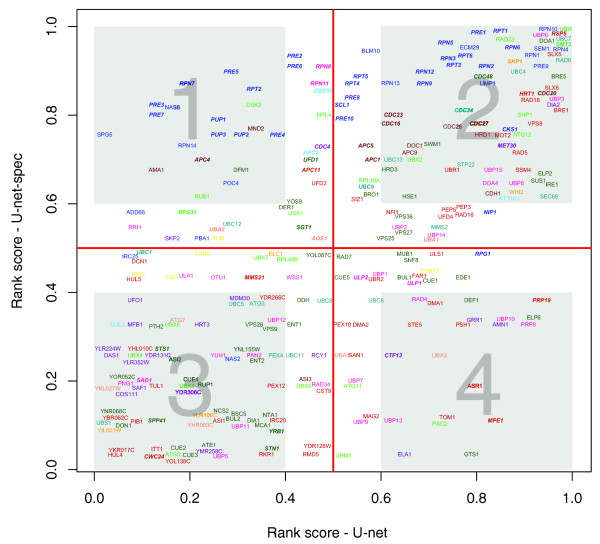
U-net components and their relative importance to the pathway and to the proteome. The figure illustrates a rank plot that reveals the presence of components of crucial importance for the U-net-specific interactions (for example, proteasome structural subunits) but not quantitatively relevant to its interaction with the proteome. On the other hand, there are other key proteins with many connections to the proteome (Ubp10 and Mpe1), but not with other Ub/Ubl pathway components. In addition, there are proteins relevant in both contexts (for example, Ubi4, Smt3, Rsp5, Rpn10), as well as proteins with just a few connections in both contexts. Gray quadrants were arbitrarily set to inspect the most important proteins in terms of degree. Essential genes are represented in bold-italic [40]. Color code: blue, proteasome components; green, Ubls; purple, F-box proteins; salmon, E1s; dark cyan, E2s; red, E3s; magenta, DUBs; dark green, others; orange, POZ; saddle brown, APC; yellow, signalosome; light blue, cullins.

To further investigate regulatory interactions of the U-net, we devised a novel visualization, the rank plot, which utilizes connectedness of a protein in both the U-net and U-net-spec along with an overlay of gene essentiality data. This plot divides the components of the Ub-system into four quadrants signifying their relative connectedness (Figure 4). The first quadrant contains proteins with a high connectivity in the U-net-spec but not in the U-net and is significantly enriched in a subset of proteasomal subunits and essential genes (FET, *P *≈ 1.54 × 10^-7^).****Most of these are core components of the proteasome, which are critical for its characteristic structure and function. This explains both their high connectivity within the U-net-spec as well as their essentiality (63%, that is, 29 out of 46 proteasome proteins are essential). The second quadrant is also enriched in proteasomal and APC proteins (FET, *P *< 0.01). These proteins have high degrees in both the U-net and U-net-spec.****In contrast to the first quadrant, the proteasomal subunits in this quadrant are responsible for recruiting modified proteins to the proteome: for example, the canonical ubiquitin receptor (Rpn10) as well as the more recently characterized second receptor, Rpn13 [42,43]. Furthermore, occurrence of the Ubl-UBA protein Rad23 in this quadrant and the significant overlap of its interactions with Rpn10 (approximately 52.6%) are consistent with the complementary and cooperative roles of these proteins [44-46]. This analysis also revealed the difference between Rad23 and its paralog Dsk2, which is found in quadrant 1 (Figure 4). Hence, Dsk2 is likely to operate on only a limited set of targets in the proteome, and might even specialize in proteins belonging to the Ub-system. Similarly, the presence of eight APC subunits in the second quadrant is indicative of the role of the APC complex in affecting a wide range of substrates in the course of cell-cycle progression (Figure 4). The DUBs Ubp6 [47] (Figure 4, quadrant 2) and Rpn11 (Figure 4, quadrant 1) are similarly discriminated, suggesting a more general role for the former in de-ubiquitinating a wide range of the proteome, whereas the latter probably acts on a smaller range of targets. Likewise, the plot illuminates the functional differentiation of several components of the U-net with comparable activities, such as the sumoylation-dependent ubiquitin ligases (Slx5-Slx8 dyad), which are in the second quadrant. This position suggests that they are not only functionally well integrated with a good part of the Ub-system but also modify a large number of target proteins. The other sumoylation-dependent E3, Uls1/Ris1, is functionally much less integrated with the rest of the Ub-system, though it might modify a similar number of targets as Slx5-Slx8. Thus, the former pair is possibly a nexus for multiple regulatory controls to influence SUMO-dependent ubiquitination. The third quadrant is enriched in F-box proteins (FET, *P *≈ 0.00135), whereas the corresponding RING finger (Hrt1) and POZ domain (Skp1) subunits of the multi-subunit E3s is found in the second quadrant. This illustrates how the distinct F-box proteins help in channeling the common RING-POZ core to distinct sets of substrates under distinct conditions.

#### Modular nature of the U-net

We then investigated the fine structure of the U-net by exploring its modular properties using two potentially complementary methods (see Materials and methods for details), the *k*-clique approach and the Markov-clustering (MCL) method. The *k*-clique approach [48,49] is an inclusive one as it allows the participation of the same protein in several cliques; it can capture the strongly interconnected elements shared between distinct biological subsystems. The MCL method [50] on the other hand restricts a protein to a single cluster, thereby bringing out the strongest functional associations in a network. The *k*-clique approach showed that the U-net contains 12,284 cliques, a number that is significantly lower than what is expected by chance alone - none of the 10,000 simulated random networks with equivalent node and edge number and degree per node ever displayed such a low number of cliques. Further, the mean degree for the U-net cliques is much lower than that observed for random networks (Wilcoxon-Mann-Whitney test (WMWT); *P *< 2.2 × 10^-16^; Table S2 and Figure S1 in Additional data file 1). We empirically observed that major hubs - for example, Ub and SUMO - co-occur in cliques much more often in the random networks (approximately 32%) compared to the real one (3.14%). These results strongly indicate that, in terms of cliques, the U-net is far less modular than equivalent random networks. The clusters resulting from the MCL method showed a distinctive size distribution: the number of clusters steadily decreases in a more or less linear fashion with increasing size till around a size of 30, followed by about 21 clusters with just a single cluster of any given size (Table S2 and Figure S1 in Additional data file 1). This again suggests that there is a strong tendency to have only few well-connected components of large-size in the U-net. Together these results indicate that both the hubs and individual modules (approximated by clusters or cliques) of the U-net are restricted in terms of their sphere of influence and tend not to display much integration between each other.

**Figure 5:**
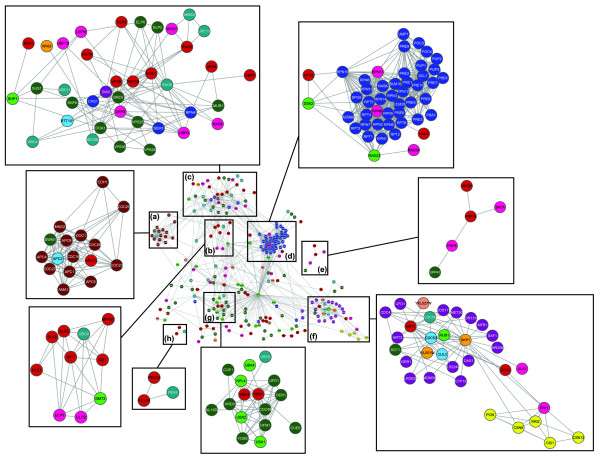
Reconstructed network using PMI. Graphical representation of the network structure captured by calculating PMI based on protein presence in cliques. Subgraphs representing important biological processes are inside boxes and magnified: APC complex (A); sumoylation pathway (B); Golgi and vesicles (C); proteasome (D); splicing (E); Skp1 and signalosome (F); ERAD (G); peroxisome (H). The colors are the same as in Figure 1. The layout of the graph to group together functionally linked dense subgraphs was achieved using the edge-weighted spring embedded (Kamada-Kawai) algorithm, which has previously been shown to be very effective for such depictions [113].

To further investigate the biological significance of cliques, we devised a novel method of identifying high-confidence functional interactions between nodes using a measure that has been termed point-wise or specific mutual information (PMI) of co-occurrence in cliques (see Materials and methods for details). We consequently identified 1,077 high confidence interactions (*P *≤ 0.005) between 258 Ub/Ubl pathway components and represented this as a graph (Figure 5; Table S2 in Additional data file 1). This graph shows a striking structure with several densely connected subgraphs that are likely to represent major functional ensembles with biological significance (Figure 4). As a positive control we checked these densely connected graphs for several previously identified complexes and found that they were faithfully recovered. Examples of these include the entire proteasomal complex with the associated DUBs and ubiquitin receptors, the signalosome, the APC complex, the ubiquitin-dependent regulatory system of peroxisomal import, and the urmylation, neddylation and sumoylation pathways. We also obtained independent corroboration for many of these linkages in the form of their co-occurrence in the clusters generated by the MCL technique.

This observation suggested that the above graph has excellent predictive potential in exploring previously under-appreciated connections when used in conjunction with sequence analysis. Here we report a few examples that are of interest in this regard. One of the densely connected regions in this graph is centered on the triad of highly connected nodes, namely the Ring finger E3 Hrt1, the POZ-domain protein Skp1 and the cullin Cdc53, which form the core of Skp1-cullin-F-box (SCF) complexes. These nodes are further linked to both the ubiquitin and Nedd8 (Rub1), the signalosome and a series of 15 F-box proteins that provide further specific links, with potential regulatory and destabilizing roles, to diverse components of both the Ub-network and the proteome. A previously uncharacterized component of this subgraph is the Ykl027w protein, which we previously identified as containing a distinctive version of the E1 domain fused to a carboxy-terminal Trs4-C domain [51]. Given that this is the only E1 superfamily protein in this subgraph, it allows us to make a functional prediction that is likely to interact with the E3 Hrt1 and the E2 Cdc34 in specific Ub/Nedd8-conjugation via certain SCF complexes. The endoplasmic reticulum (ER) associated degradation system (ERAD), which is involved in degradation or processing of proteins associated with the ER system, clearly emerges in our analysis as a distinctive subgraph. We observed that in addition to Cdc48, its target recognition receptors with Ubl domains of the Ubx family and the rhomboid-like peptidases (Der1 and Dfm1), it also includes an uncharacterized protein, Ynl155w, that is exclusively connected to this subnetwork. This protein is highly conserved in animals, fungi and amoebozoana (also laterally transferred to the apicomplexan *Cryptosporidium*) and contains an amino-terminal An1-finger combined with a carboxy-terminal SUMO-related Ubl domain. Based on its connections in the PMI graph and the presence of the Ubl domain, we predict that, analogous to the other Ubls in this system, it is likely to function as a receptor in the ERAD system that might recognize certain cytoplasmic metabolic enzymes. The significant links that we recovered between Ynl155w and the splicing factor Snu13 are also reminiscent of the earlier detected link between the splicing factor Brr2 and the ERAD system protein Sec63 [52]. This suggests that there might indeed be unexplored connections between endoplasmic protein stability and the RNA processing machinery. Examination of the PMI-derived graph in terms of connections to the rest of the proteome also helps in understanding the differentiation of certain paralogous components of the Ub-system. One case-in-point is the paralogous group of RING finger E3s, Dma1 and Dma2, which are strongly connected to each other (PMI ≈ 6.25; *P *< 10^-5^), reflecting their functional overlap in mitotic exit.****However, each of them has their own distinctive high-significance connections to the proteome: for example, Dma1 interacts with the Esc2 involved in sister-chromatid adhesion, whereas Dma2 interacts with Bub2 related to spindle orientation. Dma2 also interacts with the kinase Ime2, suggesting that it might also have a specific meiotic role [53-56].

#### Evidence for massive feedback regulation of the Ub-system

Previous studies have shown that proteasomal components are subject to possible feedback regulation via targeted modification by SCF complexes. Further, the proteasome-associated master regulator of the Ub-system, the transcription factor (TF) Rpn4 [57,58], is also extremely short lived, which is in large part due its destabilization via phosphorylation-induced ubiquitination [57,59]. This prompted us to examine if feedback regulation is a more pervasive feature of the Ub-system. To avoid conflation with generic functional interactions, we examined the self-connections in the U-net using only the specific protein-modification datasets (see Materials and methods for details). We observed that approximately 47.95% (140 out of 292) of the Ub/Ubl pathway proteins are modified by Ub and/or SUMO, the dominant modifier being Ub (42.8% of the components, FET, *P *≈ 1.54 × 10^-7^; Table S3 in Additional data file 1). While there is a slight preference for modification of proteasomal components (FET, *P *≈ 0.001), there is no significant over-representation of any particular category of proteins within the Ub-system (that is, Ubl, E1, and so on) among proteins targeted for feedback regulation. Thus, our results point to a largely unappreciated, massive post-translational self-regulation in the Ub-system at all levels. All Ub targets taken as a group did not show a lower half-life relative to non-modified proteins. This is probably due to the Ub-target set including both destabilizing K48 and non-destabilizing K63 modifications. However, our simulations showed that within the Ub-targets, modified Ub-system proteins had a notably shorter half-life than equivalently sized samples from the rest of the proteome (median *P *≈ 0.01). Hence, we suspect that this extensive self-regulation is due to destabilizing K48 modification of the Ub-system, which probably maintains the potentially destructive Ub-system under check in the cell.

### The Ub-system in the larger cellular context

#### Differential distribution of sumoylation and ubiquitination in cellular compartments

Several studies have indicated that Ub/Ubl conjugation is critical for a wide range of processes across different cellular compartments [3,60-63]. This prompted us to obtain a quantitative picture of the distribution of different modifications across compartments and also uncover any potentially novel roles for different Ub-system components in particular compartments. The most prominent difference in the relative compartment-specific distribution of modifications is with respect to sumoylation and ubiquitination. Sumoylated proteins are clearly overrepresented in the nuclear compartment (including nucleoplasm, nuclear pore, nucleolus and nuclear periphery; FET, *P *< 2.2 × 10^-16^), cytoskeleton and spindle pole, with approximately 50.3% of sumoylated proteins localized to the nucleus (Table S4 in Additional data file 1). In general, this is consistent with a well-established role for sumoylation in several processes related to chromatin dynamics, chromosome condensation, DNA repair and cell division. This process perhaps also involves interactions via the SUMO interacting motifs that are found in several nuclear proteins [64]. We observed that the highest fraction of sumoylated proteins is in the nucleolus (Table S4 in Additional data file 1), the self-organized, dynamic membrane-less subnuclear component primarily involved in biogenesis of the ribosome and several ribonucleoprotein particles [65,66]. Interestingly, the de-sumoylating peptidase Ulp1, which is anchored to the nuclear envelope via interactions with karyopherins, is absent from the envelope in regions juxtaposed to the nucleolus [3,67]. These observations are in line with prior reports showing the requirement of sumoylation for proper ribosome biogenesis [67], and specifically suggest that avoidance of de-sumoylation could be critical for structural organization of the nucleolus. An examination of sumoylated nucleolar proteins reveals that in addition to ribosome and snRNP assembly factors (for example, Nop6, Nop7, Nop8, and Nop58), multiple components of the Cdc Fourteen Early Anaphase Release (FEAR) network (for example, Cdc14, Tof2 and Fob1 [68]), are also modified.****This suggests that sumoylation could additionally be a factor in the sequestration of such regulators of replication and cell-cycle progression to the nucleolus.

In contrast, we found a significant over-representation of ubiquitination among proteins of non-nuclear compartments (FET, *P *≈ 8.86 × 10^-9^) - cell periphery, Golgi apparatus, endosomes, vesicles, vacuole and the ER (Table S4 in Additional data file 1). The cell periphery signal is likely to be enriched in Ub^K63 ^chains, which is important in internalization of membrane-associated proteins via endocytosis [60,61,69]. Further, it has been suggested that regulation of endocytosis by Ub might have a role in deciding if a particular receptor will participate in signaling or be attenuated through lysosomal degradation [69]. The well-known role of Ub, especially mono-ubiquitination, in protein sorting in the Golgi apparatus, endosomes and vesicles is consistent with the remainder of this strong non-nuclear enrichment of Ub targets.****To better understand this process, we combined these localization data with the PMI network (Figure 5) discussed above. We detected a densely connected subgraph in this network with proteins such as Bre5, Vps25 and Pep3, among others, which show predominantly Golgi-, vesicle-, and endosome-associated localization [70-72]. Interestingly, this subgraph also included the E2 ligase Rad6, which has thus far been primarily implicated in a nuclear function in mono- or poly- ubiquitination of chromatin proteins [73] and DNA replication/repair proteins [73,74]. Strikingly, two other components of the vesicular trafficking system, namely Vps71 and Vps72 and the DUB subunit Bre5, which genetically interact with Rad6, play a second role in chromatin remodeling complexes. Several members of the endosomal sorting complex required for transport (ESCRT)-II and ESCRT-III - complexes involved in vesicular trafficking - have also been implicated in RNA polymerase function and chromatin dynamics [75]. The PMI graph also hints at functional connections between different chromatin proteins and vesicular trafficking or sorting proteins (for example, Doa4 and Isw1, and Vps8 and Swr1; Table S2 in Additional data file 1). This high confidence PMI linkage of different nuclear and vesicular trafficking proteins suggests that several of these, especially those related to ubiquitin modification, might function in both cellular compartments. In particular, the suggested functional linkage of Rad6 with the cytoplasmic protein-trafficking system (Figure 5) implies that it might play a second cryptic role in this system as an E2 ligase, and might be a key component of the ubiquitinating machinery shared by the cytoplasmic and nuclear regulatory systems. It is possible that Rad6's E2 function in the cytoplasmic trafficking system is backed up by a second E2, Sec66, which has resulted in this role of Rad6 not being previously recognized in this system. Further, the results on the enrichment of ubiquitination in both the Golgi and the ER compartments emphasizes the common use of ubiquitination in the quality control of defective proteins via two very different end results - lysosomal and proteasomal degradation, respectively.

#### Regulation of chromatin proteins by the Ub-system

We then investigated interlocking between the Ub-system and nuclear processes by using a well-curated dataset of chromatin proteins [76] (Figure S2 in Additional data file 1). The signal for the specific sumoylation of chromatin proteins is very strong (FET, *P *< 2.2 × 10^-16^); even upon correcting for the general enrichment of sumoylation in nuclear proteins, we observed that chromatin proteins are specifically enriched in this modification (FET, 4.587 × 10^-7^). This observation is consistent with numerous individual observations showing a strong connection between sumoylation and chromatin functions, such as local structural remodeling as well as higher-order chromosome organization [3,5,62,77,78]. It was recently demonstrated that the SUMO-dependent Ub ligase Slx5-Slx8 associates with the DNA repair apparatus at the nuclear pore complex [79]. It was postulated that sumoylated proteins might accumulate at collapsed forks or double-strand breaks, thereby requiring proteasomal degradation due to Slx5-Slx8 mediated ubiquitination for their clearance. Pol32, Srs2 and Rad27 were suggested as potential targets for such a degradation process [79].****Consistent with this proposal, all these genes were recovered as interacting with Slx5-Slx8 in our PMI network. Moreover, we also identified several other genes as part of this densely connected subgraph of the PMI network (Figure S2 in Additional data file 1) with a potential role in DNA repair. Of particular interest in this regard is the tyrosyl-DNA-phosphodiesterase (Tdp1), which localizes to single-stand breaks with covalently linked DNA-topoisomerase linkages [80], and Rad9, a component of the 9-1-1 complex [81]. These observations suggest that such SUMO-dependent targeting of proteins might additionally be critical for clearing proteins accumulated at single-strand breaks and other DNA lesions sensed by the 9-1-1 complex.

A study of the PMI graph (Figure 5) in conjunction with evolutionary conservation patterns also helped us predict a key role for Sus1 in coordinating different Ub modification events of chromatin proteins. Sus1 is predicted to form a 4-helical bundle (File S2 in Additional File 1) and earlier studies have shown that it is associated with the nuclear pore, as part of the minimal histone H2B de-ubiquitinating complex in conjunction with the DUB Ubp8. We also found that plants contain a second paralog of Sus1 (File S2 in Additional File 1) that is fused to the carboxyl terminus of another DUB (Ubp25 [GI:30688637]), suggesting a conserved functional association between Sus1 and de-ubiquitination. Our analysis of the evolutionary conservation patterns of components of this complex showed that whereas Sg11 (with a Rad18 finger) and Sg73 (with a CCCH finger) are restricted to the eukaryotic crown group, Sus1 itself is found in kinetoplastids as well as parabasalids. This indicates that Sus1 was present in the last eukaryotic common ancestor and implies that it is the primary conserved subunit of the histone H2B de-ubiquitinating complex. The PMI graph shows that SUS1 also shows significant functional links to two E2 ligases, Ubc11 and Ubc4, as well as the E3 Ris1/Uls1. These associations suggest that in addition to being a subunit of the DUB, Sus1 might also recruit E2s or an E1 and thereby function as a common adaptor for both chromatin protein ubiquitination and de-ubiquitination.

#### Interplay between the ubiquitin system and transcription

We combined the comprehensive transcriptional network compiled earlier by our group [34,39] with the U-net presented in this study to examine the functional interplay between these two major regulatory systems in the cell. We observed that in addition to the activator of proteasomal genes Rpn4 (FET, *P *< 2.2 × 10^-16^) and Reb1 (FET, *P *≈ 0.0002) [34], there are few other potentially significant regulators of the Ub-system (FET, *P *< 0.015; Table S5 in Additional data file 1), namely Aft1, Sip4 and Yap3. Examination of other targets, which are likely to be co-regulated with the Ub-system genes by these TFs, indicates possible conditions or aspects of cellular metabolism in which they might be involved: Aft1 targets appear to be generally linked to iron metabolism [82], Sip4 targets are related to gluconeogenesis [83] and Yap3 targets are involved in stress response [84]. In terms of incoming connections of TFs to components of the Ub-system (that is, number of regulatory inputs from TFs to Ub-system genes) we observed no obvious relationship between connectedness of a given gene in the U-net and its inputs from the T-net (Table S5 in Additional data file 1). Hence, more tightly regulated genes do not necessarily have more interacting partners or a wide range of distinct functions. However, certain genes are highly regulated by a large number of TFs and might be required in multiple distinct conditions. The Ub-system gene with the highest number of inputs is the uncharacterized F-box protein- encoding gene YMR258C (16 different input TFs). Based on it is interaction partners (Aro1, Faf1, Ypt52, Adh1, Gdh1, Hsp82, Gdi1, Ymr1), most of which are ubiquitinated, it is predicted to participate in diverse processes such as carbohydrate metabolism, vesicular trafficking and RNA processing. Hence, depending on the transcriptional input, the same E3 subunit might be potentially reused in very distinct functional contexts. SUMO and Nedd8 (Rub1) also receive a higher than typical number of TF inputs (ten TFs), suggesting that these modifiers might be controlled at the transcriptional level by a diverse set of stimuli. Thus, specific transcriptional regulation of different Ub-system genes appears to enable them to be reused to regulate different cellular processes.

From the reverse perspective, one third of all TFs in the T-net are ubiquitinated and/or sumoylated (Table S5 in Additional data file 1). The fraction of sumoylated TFs is not significantly different from the fraction of sumoylated TFs in the nuclear proteome, suggesting that unlike chromatin proteins, there is no preferential sumoylation of TFs beyond the nuclear background.****Ubiquitination was, however, generally underrepresented amongst TFs with respect to both the whole proteome (FET, *P *≈ 0.006) and also just the nuclear proteome (FET, *P *≈ 0.018). Despite this trend, we observed that ubiquitinated TFs tended to have a higher number of significant co-regulatory interactions with other TFs (that is, significant sharing of target genes with other TFs, see [34,39] for details; *P *< 0.0001). Based on these observations, it appears that ubiquitination of TFs, while less frequent, might have a specific role in influencing their co-regulatory interactions. The low incidence of TF ubiquitination also questions the role of ubiquitination in modulation of TFs by degradation. On the whole, Ub and SUMO might exert a considerable biological influence via transcription regulation because TFs modified by them together regulate 2,899 genes, which is nearly half of the proteome.

#### Interplay between cell cycle-linked gene expression and control via the Ub-system

We further explored the link between gene expression and the Ub-system to investigate if there was any interplay between Ub/Ubl modifications and variations in gene expression over the cell cycle. Using data published by Spellman *et al*. [85], we compiled a list of genes whose expression varied periodically over the progression of a cell cycle and checked their products for post-translational regulation by Ub/Ubl modification (Table S6 in Additional data file 1). Interestingly, products of these cyclically expressed genes showed a certain propensity for being preferentially ubiquitinated (FET, *P *≈ 0.002) but not sumoylated. We also uncovered a certain propensity for genes induced by cyclins Cln3 and Clb2 [85] to be preferentially ubiquitinated (FET, *P *≈ 0.007). Thus, in addition to regulation at the level of gene expression, the products of a subset of these genes with periodic expression over the cell cycle might experience a potentially reinforcing post-translation regulation by means of ubiquitination. Interestingly, while the products of genes regulated by Clb2 showed a tendency not to be sumoylated, products of those regulated by Cln3 showed some preference for sumoylation (for example, histones, cohesin and cytoskeletal components; FET, *P *≈ 0.018). Thus, in contrast to ubiquitination with its general role in regulation of protein levels, the interplay between sumoylation and cyclic gene expression might have a specific role in the assembly of certain nuclear and cytoskeletal complexes linked to the G1 phase of the cell cycle.

#### Similarities and differences in the properties of targets of Ub and SUMO modification

We then systematically investigated different gross properties of Ub and SUMO targets to understand their general cellular properties and the implications thereof. For this purpose we integrated the modification dataset with the large-scale datasets for protein abundance [30,31], half-life [29] and frequency of optimal codons [86]. Both ubiquitinated and sumoylated proteins have higher abundances than unmodified proteins (WMWT, *P *< 2.2 × 10^-16 ^and *P *< 0.01, respectively; Figure S3 in Additional data file 1), with proteins undergoing both modifications showing even higher abundances (WMWT, *P *< 2.2 × 10^-16^). In agreement with their higher abundances, ubiquitinated and sumoylated proteins show a significantly higher frequency of optimal codons and appear to be more efficiently translated than non-modified proteins (WMWT, *P *< 2.2 × 10^-16 ^and *P *≈ 1.22 × 10^-9^, respectively; Figure S3 in Additional data file 1). While one could posit a technical bias towards detection of abundant proteins, the use of sensitive mass spectrometry methods to detect even rare species suggests that this might not be a major confounding factor. Based on these observations, it appears that regulation via conjugation of protein modifiers predominantly targets abundant and efficiently translated proteins. However, given the divergence in the roles of sumoylation and ubiquitination, it is likely that the effects on their targets are very distinct. For example, we found that ubiquitinated proteins, but not sumoylated proteins, show a lower half-life than their unmodified counterparts (Figure S3 in Additional data file 1). However, this difference is not strong (WMWT, *P *≈ 0.03), which is in apparent contradiction to the powerful Ub-dependent proteasomal degradation activity. However, there are two possible explanations for this observation, which are not mutually exclusive: first, the ubiquitination datasets do not distinguish between the predominantly destabilizing K48 polyubiquitination on the one hand and the K63 polyubiquitination and monoubiquitination on the other, which have no destabilizing effects; and second, protein levels can rapidly become undetectable after Ub-tagging, and these abrupt changes in protein levels might not be captured by the traditional half-life estimations involving antibodies or green fluorescent protein-tagged constructs.

We also examined the relationship between Ub or SUMO modification and the presence of low complexity regions (LCRs), which are repetitive or unstructured regions frequently found in eukaryotic proteins (Figure S3 in Additional data file 1). Sumoylated and/or ubiquitinated proteins have higher fractions of LCRs (WMWT, *P *≈ 6.01 × 10^-10^), with sumoylated proteins having even higher fractions of LCRs than their ubiquitinated counterparts (WMWT, *P *≈ 6.71 × 10^-9^). It was previously hypothesized that hubs in the protein network tend to have higher fractions of amino acids spanning LCRs and a role in protein-protein interactions was proposed [87]. However, we did not observe a straightforward positive correlation between the LCR content and degree of a given protein in the U-net; hence, the earlier reported observation might be an artifact of the presence of spuriously 'sticky' LCR-rich 'hubs' in the protein-protein interaction network. On the other hand we did find a striking prevalence for both ubiquitination and sumoylation among hubs (FET, *P *< 2.2 × 10^-16^). Enrichment in ubiquitination perhaps reflects a targeted control of hubs through degradation by the ubiquitin-proteasome system. As nuclear proteins in general are enriched in hubs, we then tested if enrichment of sumoylation in hubs might merely be a consequence of that observation. Even after correcting for this nuclear enrichment of hubs we found a clear propensity for sumoylation among hubs (FET, *P *≈ 5.27 × 10^-5^). Thus, sumoylation could potentially serve as a platform for allowing secondary interactions through SUMO-interacting motifs and increase the total number of interactions of a protein. Thus, it appears that both modifications tend to preferentially target abundant proteins and hubs, but appear to exert distinct influences on their targets; Ub probably has a role in destabilizing its targets, whereas SUMO probably contributes to increased number of interactions.

### Evolutionary implications of the reconstructed network

A precise understanding of how the U-net has diversified in the course of evolution requires comparable networks from other eukaryotes. Although there have been several recent datasets that provide information to allow limited reconstructions in other eukaryotes, we feel that the data are still vastly insufficient to attempt any meaningful comparison with the current network available for *S. cerevisiae*. However, analysis of the conservation patterns of nodes and the general structure of this *S. cerevisiae *network does throw light on both the early diversification of the Ub-system as well as some general evolutionary trends of particular components. Our earlier investigation of the evolution of Ub/Ubls in eukaryotes and other Ub-like proteins suggests that eukaryotes probably acquired the basic precursors of the Ub conjugation system, like the ancestral E1 and E2 enzymes, from a bacterial source [13,88]. Given that there are no extant primitively amitochondriate eukaryotes, the most parsimonious scenario would imply that this bacterial source was the progenitor of the mitochondrion [89]. From the time of this first eukaryotic common ancestor with the bacterial endosymbiont to the last eukaryotic common ancestor (LECA) of all extant lineages there was an explosive radiation of the Ubl superfamily resulting in several conjugated and non-conjugated forms [51]. It is likely that the ancestral conjugated form had a general role of a tag in the degradation of targeted proteins because peptide tagging (for example, tmRNA-derived peptides and pupylation in bacteria [90-92]) has been an ancient solution to the problem of specifying proteins for unfolding and degradation by different ATP-dependent proteolytic systems. However, the explosive early radiation of the Ubl superfamily suggests that even before LECA these tags appear to have been utilized in contexts other than degradation, such as specific protein-protein interactions.

Our current analysis of the U-net helps in understanding the context of differentiation of the primary conjugated forms, Ub and SUMO. We observed a strong signal for the preferential nuclear enrichment of SUMO compared to the cytoplasmic enrichment of Ub, especially in the context of vesicular, vacuolar and ER complexes. Even the SUMO E3s show a predominantly nuclear localization and nuclear interaction partners (Figure S3 in Additional data file 1). This suggests that the divergence of Ub and SUMO was probably correlated and coeval with the emergence of the nucleus as a separate compartment from the cytoplasmic ER network. SUMO probably acquired a dominant nuclear role while Ub a dominant cytoplasmic role. Previously, sumoylation has been shown to exhibit a preference for lysine occurring in the signature sequence hxK [ED] (where h is a hydrophobic residue and x any residue) [93]. However, it was not clear if the Ub sites exhibit any preference at all. We utilized the dataset identifying the individual modified lysines on Ub targets [22] and 1,000 randomly picked lysines as a comparison for statistical purposes to investigate if there was any preference in the Ub modification site. We noticed a preference for a motif of the form [ED]Kx4 [ED] spanning the modified lysine, and a mild general enrichment for acidic residues for around five positions on either side of the modified K (Figure S4 in Additional data file 1). This suggests that in addition to divergence of the modifiers, SUMO and Ub themselves, even their target site preferences differentiated to a certain extent. Consistent with this, the E1, E2 and E3 enzymes for Ub and SUMO appear to have diverged considerably in the interval between the first eukaryotic common ancestor and LECA, with distinct SUMO- and Ub-specific E3s by the time of LECA. Further, specific nucleolar enrichment and function suggest that the divergence of SUMO might be related to the emergence of this key subcompartment within the nucleus.

Phyletic patterns of SIM-containing SUMO-dependent Ub E3s reveal an interesting pattern: apparently, Rnf4 (Slx8) orthologs are conserved throughout the eukaryotic crown group (animal, fungi, amoebozoans and plants) and have been transferred to chromists from their plant symbiont. However, Slx5 (Rfp1 and Rfp2 in *S. pombe*) is restricted to the ascomycete fungi and appears to have emerged in that lineage through a duplication of Slx8. The other potential SUMO-dependent E3, Ris1 (Uls1), is also restricted to the eukaryotic crown group. These observations would suggest that the functional linkage between sumoylation and ubiquitination was a relatively late phenomenon. However, it cannot be ruled out that other eukaryotes possess uncharacterized SUMO dependent Ub ligases. In this light it is interesting to note that Rad5 (a more ancient Ris1 paralog) shows strong functional links in the PMI network with different SUMO pathway proteins, namely Ubc9 (the SUMO E2) and Wss1 (a potential SUMO DUB). Hence, it would be of interest to investigate if Rad5 might have SUMO-dependent ubiquitination activity.

Examination of our reconstructed network in light of the conservation patterns of components of the ER associated ubiquitination system also throws light on the origin of the ERAD system. Within the core ERAD system identified here through PMI analysis (Figure 5) specific components, such as the ATP-dependent unfoldase Cdc48, the key Ub-interacting protein Npl4, the UBX and CUE domains of receptors of targeted proteins, and the rhomboid-like peptidase (Dfm1 and Der1) [94], can be traced back to LECA. Of these, Cdc48 can be traced to the archaeal component of the eukaryotic progenitor and the rhomboid-like peptidase Dfm1/Der1 to the bacterial symbiont. In archaea, Cdc48 homologs function as chaperones in association with the RNA-degrading exosome or as chaperones of membrane proteins [95,96]. Multiple eukaryotic cytoplasmic complexes, such as the ribosome, the T-complex chaperone and the core of ESCRT-III, have an archaeal origin, suggesting that many complexes functioning in the cytosol of the archaeal progenitor of eukaryotes were directly inherited by the eukaryotic cytoplasm [89]. In a similar fashion it is possible that Cdc48, which was associated with the cytosol and the membrane of the archaeal progenitor, was retained as the core of a key ER membrane associated chaperone system in eukaryotes. However, the elaboration of this system proceeded very differently in eukaryotes, with rhomboid peptidases acquired from a bacterial endosymbiont being recruited as new components that were critical in the context of an internal membrane - the ER. The remaining components were eukaryotic innovations; two of them - the UBX domain and the novel Ubl in Npl4 - emerged as part of the early eukaryotic radiation of the Ubl superfamily [51]. The CUE domain appears to have been part of the radiation of Ub-binding domains of the UBA-like fold, whereas the inactive Jab domain in Npl4 is a part of the notable radiation of active as well as inactive Ub-binding Jab domains in early eukaryotic evolution [51,97]. The Zn-clusters in Npl4 appear to be *de novo *innovation of a Zn-supported eukaryote-specific structure. Thus, the early radiation of Ubls and Ub-associated domains provided a new 'eukaryotic' layer that connected the ancient membrane-linked chaperone system to the incipient Ub-system. Similar recruitment of Ub-system components to the ESCRT-III complex, inherited from archaea, appears to have been central to the emergence of the new role of the eukaryotic ESCRT complex in vesicular trafficking, in addition to its ancestral function in cell-division [98,99].

Our earlier study of lineage-specific expansions and innovations in the Ub-system showed that while E1 and E2 are largely vertically inherited over eukaryotic evolution, the E3s and their subunits, and to a lesser extent the DUBs, are subject to numerous lineage-specific expansions or innovations [100]. This pattern was explained on the basis of the core structure of the conjugation pathway, in which a common stem comprising E1 and E2 is recruited to a very diverse set of targets by means of E3s and their subunits. Similarly, lineage-specific innovations in DUBs are seen as driven by a need to accommodate larger substrate diversity. An examination of our PMI-based network shows that one of the most striking dense subgraphs is centered on the Skp1, Hft1 and Cdc53 (Figure 5). These are in turn connected to numerous F-box proteins in a 'star-like' topology. This organization suggests that with a relative small set of RING finger E3 ligase and cullin subunits a great diversity of SCF E3s is achieved, primarily via the multiplicity of F-box subunits. Interestingly, the largest independent lineage-specific expansions in the Ub-system are seen in F-box proteins (for example, plants and nematodes), POZ (BTB) and MATH domain proteins (which take the place of the POZ domain Skp1 in the SCF complexes; for example, plants and different animals), both of which are components of SCF. The organization of the SCF subgraph of the above network suggests that this organizational principle has probably favored repeated lineage-specific diversification of the SCF complex widely across different eukaryotes. Such lineage-specific expansions were previously suggested to have a role in pathogen response; hence, these SCF complexes might have independently radiated in different eukaryotic lineages as a part of the intracellular immune system that recognizes a diversity of intracellular pathogens and degrades their proteins [101].

## Conclusions

By reconstructing the first comprehensive network representation of the Ub pathway for a model eukaryote, we were able to investigate for the first time the Ub-system not merely in terms of individual components but as a whole. As a result we were able to obtain a quantitative picture of how different subsystems interact within the Ub-system and develop an understanding of the diversification of the biochemistry of paralogous and functionally analogous components of the system. We also developed a novel point-wise mutual information based method that helps in assessing strengths of particular functional connections in the network and delineating the most relevant interactions. The reconstruction also helped us recover new connections that have predictive value regarding previously poorly understood components (for example, of SCF-based ubiquitination and ERAD) and might be of use in further experimental investigation. Finally, we were also able to estimate the extent of interlocking between other major regulatory systems such as transcription and the ubiquitin system and the compartment-specific diversity in modification by ubiquitin-like modifiers. We also use the structure of the network reconstructed here to understand certain key tendencies observed in the evolution of the ubiquitin system. We hope the model presented here will provide a platform not only for integrating distinct datasets but that also allows comparisons between different eukaryotes in the future.

## Materials and methods

### Defining the Ub-system components, datasets and network assembly

The Ub/Ubl system proteins used in our reconstruction are manually curated and frequently updated via extensive literature mining as presented in earlier publications by our group [13,51,102]. For assembling a comprehensive interaction map using publicly available data we used the following databases: BioGRID (version 2.0.45) [103,104], IntAct (version 02/10/2008) [105] and MINT (version 5/16/2008, without computationally predicted interactions) [106]. These were used to build the interaction network, which was further complemented by specific ubiquitination [19-23], sumoylation [24-28], Rsp5 (E3 ligase) [33] and the proteasome subunit Rpn10 data [20,21]. All data processing was locally performed with custom scripts using the open reading frame identifiers from the *Saccharomyces *Genome Database [86]. To assemble the Ub network (graph), all pair-wise interactions (edges) that involved at least one component of the Ub/Ubl pathway (described in the previous section) were used. We have also assembled a protein-protein interaction network by filtering this type of interaction from BioGRID[103], IntAct [105] and PMINT [106]. All analyses of ubiquitination and sumoylation mentioned in the text were performed using only the Ub/SUMO-specific datasets mentioned above.

Other datasets used in this study include: environmental gene essentiality [40]; genomic profiling [41]; protein half-life, localization and abundance [29-32]; chromatin- and cell cycle-related proteins [76,85]. The environmental genomic profiling dataset is composed of genes important to normal growth under medium and/or nutrient changes (environmental) and chemical stresses (exposure to small molecules). Only the first category was used here. We define a gene as involved in environmental stress response if it reached statistical significance (*P *≤ 0.001) in at least 80% of the replicates in the original dataset [41]. As many high-throughput datasets are not readily available through public databases and/or in plain text formats with unique identifiers, their integration and analysis necessitated extensive case-specific data extraction through literature searches, reorganization and collation via custom Perl scripts.

### Data processing, statistical testing and simulations

Basic network analyses were carried using Perl scripts [107] and all statistical tests were performed using the R statistical language [108]. For simulation purposes, 10,000 random networks were created by re-wiring the edges of the original network using a previously described strategy [109], maintaining the original degree of each node. In assessing robustness of the U-net to attack/failure [38,110], edges created due to a link with Ub or SUMO interactions were excluded to avoid biases due to these major hubs. The logo representation of the ubiquitination sites was plotted using WebLogo [111]. Graphs were rendered using Cytoscape [112].

### Assessing network modularity

In the *k*-clique approach we identified complete subgraphs with *k*-vertices using two independent programs that produced identical results [48,49]. Incomplete (or defective) cliques [49] were also generated via merger of cliques into larger modules, annotated and analyzed [34]. Merger of cliques with at least 51% overlapping nodes resulted in 12,284 cliques generating 574 modules. For MCL we used the unsupervised clustering implemented in the MCL package [50]. We assigned weights for the interactions using simple topological overlap between two nodes and used the resulting weighted graph for computation of clusters with the MCL program [47]. We then identified high-confidence interactions involving different proteins of the U-net using a novel approach of point-wise mutual information. PMI is effectively a measure of the association between two nodes *i *and *j *in the network by using their joint distribution (p(*i, j*)) and the product of their marginal distributions (p(*i*) and p(*j*), respectively):

(1)

To assess the significance of the PMI value between two nodes we computed cliques for 10,000 random networks and calculated the *p*-value for a pair of nodes as the fraction of the random networks that presented the PMI score of at least the same value as the U-net.

## Abbreviations

DUB: de-ubiquitinating peptidase; ER: endoplasmic reticulum; ERAD: endoplasmic reticulum associated degradation system; ESCRT: endosomal sorting complex required for transport; FET: Fisher exact test; LCR: low complexity region; LECA: last eukaryotic common ancestor; MCL: Markov-clustering; PMI: point-wise mutual information; P-net: protein-protein network; SCF: Skp1-cullin-F-box; TF: transcription factor; T-net: transcriptional regulatory network; Ub: ubiquitin; Ubl: Ub-like polypeptide; U-net: ubiquitin network; U-net-spec: Ub specific network; WMWT: Wilcoxon-Mann-Whitney test.

## Authors' contributions

TMV and LA conceived the study, analyzed the results and wrote the paper. TMV implemented the computational methods and integrated the public datasets. SB and LMI contributed high-quality data and ideas and helped in preparing the final version of the manuscript, which was read and approved by all the authors.

## Additional data files

The following additional data are available with the online version of this paper: a zip file including Tables S1-S7, Figures S1-S4 and Files S1 and S2 (Additional data file 1)

## Supplementary Material

Additional data file 1 Table S1: annotations and additional information on all the U-net components. Table S2: modular structure of the U-net. Table S3: feedback regulation of the Ub/Ubl pathway. Table S4: ubiquitination/sumoylation and cellular localization. Table S5: Ub/Ubl pathway and transcription factors. Table S6: cell cycle-related proteins modified by Ubls. Table S7: interactions of the Slx5-Slx8 complex in the MI network. Figure S1: clique degrees and sizes in the U-net and random networks. Figure S2: chromatin proteins regulated by Ub and SUMO. Figure S3: properties of ubiquitinated and sumoylated proteins. Figure S4: logo representation of the flanking regions of ubiquitinated lysines. File S1: plain text representation of the U-net. File S2: multiple sequence alignment of the SUS1 domain. Click here for file
