# Supplementary material for: Reconstructing the ubiquitin network - cross-talk with other systems and identification of novel functions
Source: Genome Biol. 2009 Mar 30;10(3):R33. doi: 10.1186/gb-2009-10-3-r33 (PMC2691004; doi:10.1186/gb-2009-10-3-r33)
Supplement: Additional data file 1 — Table S1: annotations and additional information on all the U-net components. Table S2: modular structure of the U-net. Table S3: feedback regulation of the Ub/Ubl pathway. Table S4: ubiquitination/sumoylation and cellular localization. Table S5: Ub/Ubl pathway and transcription factors. Table S6: cell cycle-related proteins modified by Ubls. Table S7: interactions of the Slx5-Slx8 complex in the MI network. Figure S1: clique degrees and sizes in the U-net and random networks. Figure S2: chromatin proteins regulated by Ub and SUMO. Figure S3: properties of ubiquitinated and sumoylated proteins. Figure S4: logo representation of the flanking regions of ubiquitinated lysines. File S1: plain text representation of the U-net. File S2: multiple sequence alignment of the SUS1 domain. [file gb-2009-10-3-r33-S1.zip › addFile1/index.html]

**Reconstructing the ubiquitin network – cross-talk with
other systems and identification of novel functions**

 

Thiago M Venancio\*, S Balaji,
Lakshminarayan M Iyer and L Aravind\*

 

 

National
Center for Biotechnology Information, National Library of Medicine, National
Institutes of Health, Bethesda, Maryland 20894, USA

 

\*Correspondence:
{venancit, aravind}@ncbi.nlm.nih.gov

 

**ABSTRACT**

 

**Background**

The
ubiquitin system (Ub-system), a quintessential feature of eukaryotes, can be
defined as the ensemble of components including Ub/ubiquitin-like proteins,
their conjugation and deconjugation apparatus, binding partners and the
proteasomal system. While several studies have concentrated on structure-function
relationships and evolution of individual components of the Ub-system, a study
of the system as a whole is largely lacking.

**Results**

Using
numerous genome-scale datasets, including protein-protein- and genetic-
interactions and gene expression data, we assemble for the first time a
comprehensive reconstruction of the budding yeast Ub-system. By representing it
as an undirected graph (the U-net) we were able to carry out a number of
operations on it and decipher its static and dynamic properties. We also
devised two novel representations, namely the rank plot to understand the
functional diversification of different components and the clique-specific point-wise
mutual-information network to identify significant interactions in the Ub-system.

**Conclusions**

By
means of these analyses we show that the U-net is more susceptible to attack
than the transcription regulatory network or the general protein-protein
interaction network and could thus be a relatively vulnerable element in the
cell’s physiology. We were also able to use the rank-plot to understand the
functional diversification of various components such as different
ubiquitinated protein receptors and SUMO-dependent Ub-ligases. Using the mutual
information network we were able to predict novel components of certain Skp1-cullin-F-box
(SCF)-dependent ubiquitination pathways, receptors in the endoplasmic reticulum
associated degradation system (ERAD) system and a key role for Sus1 in
coordinating multiple Ub-related processes in chromatin dynamics. We also
present evidence for the Ub-system exerting a major indirect regulatory role on
large parts of the proteome via its interaction with the transcription
regulatory network. Further analysis of the dynamics of the U-net suggests that
Ub and SUMO modifications might function cooperatively with transcription
control in the regulation of cell-cycle-stage-specific complexes and in reinforcing
periodicities in gene expression. Finally, when combined with evolutionary
information derived from conservation patterns, the structure of the U-net
helps in understanding better the lineage-specific expansion of SCF complexes
with a potential role in pathogen response and the origin of systems such as
ERAD and the endosomal sorting complex required for transport (ESCRT).

**CONTENTS:**

 

·        
File
S1 - Plain text representation of the
U-net.. 2

·        
Table
S1 - Annotations
and additional information on all the U-net components.  2

·        
Figure
S1 - Cliques
degrees and sizes in the U-net and random networks.. 2

·        
Table
S2 - Modular
structure of the U-net.. 3

·        
Table
S3 - Feedback
regulation of the Ub/UBL pathway.. 3

·        
Table
S4 -Ubiquitination/Sumoylation
and cellular localization.. 3

·        
Figure
S2 - Chromatin
proteins regulated by Ub and SUMO.. 3

·        
Table
S5 - Ub/UBL
pathway and transcription factors.. 3

·        
Table
S6 - Cell
cycle related proteins modified by UBLs.. 3

·        
Figure
S3 - Properties
of ubiquitinated and sumoylated proteins.. 3

·        
Figure
S4 - Logo
representation of the flanking regions of ubiquitinated lysines.  9

·        
File
S2 - Multiple
sequence alignment of the SUS1 domain.. 10

·        
Table
S7 - Interactions
of the Slx5-Slx8 complex in the MI network.. 11

 

## Plain text representation of the U-net.

                Tabular
representation of the interactions in the U-net. ORF names were used to define
each node of the graph.

 

## Annotations of all the U-net components.

Annotation of each
gene in the U-net and its respective annotation, sumoylation and ubiquitination
status. For simulation purposes, we created a modified U-net, excluding the
connections involving Ub and SUMO. By visually inspecting the inflexion point
of the degree distribution of such network we defined 305 most connected nodes
as the hubs of the U-net. All the components of the U-net, along with their
annotations can be found here.

## Cliques degrees and sizes in the U-net and random networks.

**A-** Box plot representation illustrating the lower average
clique degree distribution of the U-net (green) when compared to the random
networks (red) (WMWT; *p* < 2.2e-16).
**B-** Distribution of clique sizes,
showing a tendency for larger cliques in random networks, which is a
consequence of the connections between high-degree nodes.

 

 

## Modular structure of the U-net.

          Merged cliques.

          Markov clusters.

          Mutual information.

## Feedback regulation of the Ub/UBL pathway.

Tabular representation of the Ub/UBL
pathway components with their respective sumoylation and ubiquitination status.
Ub is the main player in this regulatory process (FET, *p* = 1.54e-7). Overall, ~47.95% (140/292)Ub/UBL pathway
components are ubiquitinated.

## Ubiquitination/Sumoylation and cellular localization.

The results are presented in three
different ways: total counts, compartment and modification perspectives.

## Chromatin proteins regulated by Ub and SUMO.

We found a striking enrichment of
sumoylation targets among the chromatin proteins previously classified by our
group (FET, *p* < 2.2x10^-16^),
a trend that is maintained when considering only nuclear proteins (FET, 4.59x10^-7^),
suggesting that its nature is not only a due to the nuclear enrichment of SUMO
targets. A visual representation is presented below. The color code is the same
as used in figure 2 and 3 of the manuscript. A SVG version of the figure is
also provided here.

 

 

 

 

## Ub/UBL pathway and transcription factors.

Here we show the relations between TFs and
Ub/UBL pathway that 190 of 292 (~65%) pathway components are regulated by at
least one TF. The major TFs in terms of Ub/UBL pathway regulation are also
shown. In addition to the previously reported Rpn4p and Reb1p, we also
identified three other important TFs (p < 0.015), namely Aft1p, Sip4p and
Yap3p.

## Cell cycle related proteins modified by UBLs.

Relation between  periodically expressed genes with
ubiquitination/sumoylation. A slight preference for ubiquitination among
periodically expressed genes (FET, *p*
= 0.002) were found. This trend is not observed for sumoylation. In addition,
different patterns of ubiquitination and sumoylation among genes activated by
the cyclins Cln3 and Clb2 were also observed, as discussed in the paper.

## Properties of ubiquitinated and sumoylated proteins.

Sumoylated and ubiquitinated proteins are
compared in terms of their abundance, half-life, frequency of optimal codons,
length, degree and fraction of low complexity regions (see methods for
references).

## Logo representation of the flanking regions of ubiquitinated lysines.

There is a clear trend favoring acidic
amino acids neighboring the targeted lysine. Flanking regions were fetched from
the proteome using custom scripts.

 

 

 

## Multiple sequence alignment of the SUS1 domain.

Each protein is denoted by
its gene name, species abbreviation and genbank index number (GI) separated by
underscores. Secondary structure was predicted using the JPRED program and is
shown above the alignment where H refers to an amino acidposition predicted to
be in a helix. The alignment conservation was calculated at 90% consensus and
isshown below the alignment. Consensus abbreviations are as follows: s: small
residues (ACDGNPSTV; u:tiny residues (GAS);p: polar residues (CDEHKNQRST);b:
big residues (KMILEWRYFQ);l: aliphatic residues (LIV);c: charged residues
(DEHKR); h: hydrophobic residues (ACFILMVWY). Species abbreviations are as
follows: Aaeg : Aedes aegypti; Agos : Ashbya gossypii; Amel : Apis mellifera;
Apis : Acyrthosiphon pisum; Atha : Arabidopsis thaliana; Bmal : Brugia malayi;
Calb : Candida albicans; Cbri :Caenorhabditis briggsae; Ccin : Coprinopsis
cinerea; Cele : Caenorhabditis elegans; Cgla : Candida glabrata; Cint : Ciona
intestinalis; Cneo : Cryptococcus neoformans; Cqui : Culex quinquefasciatus;
Crei : Chlamydomonas reinhardtii; Ddis : Dictyostelium discoideum; Dhan :
Debaryomyces hansenii; Dmel : Drosophila melanogaster; Drer : Danio rerio; Ggal
: Gallus gallus; Hsap : Homo sapiens; Klac :Kluyveromyces lactis; Lbic :
Laccaria bicolor; Lelo : Lodderomyces elongisporus; Linf : Leishmania infantum;
Lmaj : Leishmania major; Mbre : Monosiga brevicollis; Mdom : Monodelphis
domestica; Mmul : Macaca mulatta; Mmus : Mus musculus; Nvec : Nematostella
vectensis; Nvit : Nasonia vitripennis; Oana : Ornithorhynchus anatinus; Osat :
Oryza sativa; Pgui : Pichia guilliermondii; Ppat : Physcomitrella patens; Pram
: Phytophthora ramorum; Psit : Picea sitchensis; Ptet : Paramecium tetraurelia;
Ptri : Phaeodactylum tricornutum; Ptro : Pan troglodytes; Rnor : Rattus
norvegicus; Scer : Saccharomyces cerevisiae; Spom : Schizosaccharomyces pombe;
Spur : Strongylocentrotus purpuratus; Tadh : Trichoplax adhaerens; Tbru :
Trypanosoma brucei; Tcas : Tribolium castaneum; Tcru : Trypanosoma cruzi; Tgon
: Toxoplasma gondii; Tgut : Taeniopygia guttata; Tnig : Tetraodon nigroviridis;
Tthe : Tetrahymena thermophila; Tvag : Trichomonas vaginalis; Umay : Ustilago
maydis; Vvin : Vitis vinifera; X(Si : Xenopus (Silurana); Xlae : Xenopus
laevis; Ylip : Yarrowia lipolytica; Zmay : Zea mays.

 

## Interactions of the Slx5-Slx8 complex in the MI network.

The complex is postulated
to be involved in DNA repair. We recovered its interactions in the MI network.
The putative targets previously proposed by Nagai et al. are represented in
yellow. We also suggested additional targets (blue).
