# Supplementary figures and images for: Reconstructing the ubiquitin network - cross-talk with other systems and identification of novel functions
Source: Genome Biol. 2009 Mar 30;10(3):R33. doi: 10.1186/gb-2009-10-3-r33 (PMC2691004; doi:10.1186/gb-2009-10-3-r33)

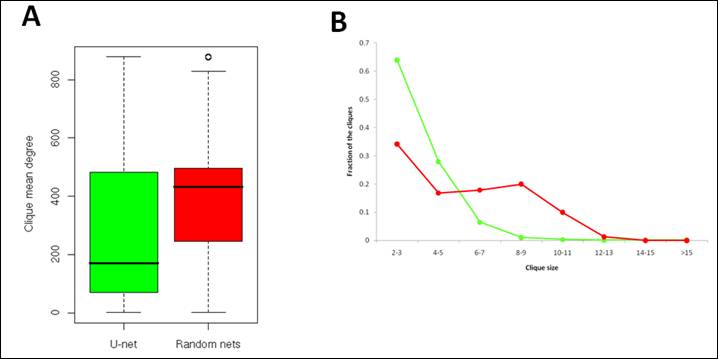

Supplement: Additional data file 1 — Table S1: annotations and additional information on all the U-net components. Table S2: modular structure of the U-net. Table S3: feedback regulation of the Ub/Ubl pathway. Table S4: ubiquitination/sumoylation and cellular localization. Table S5: Ub/Ubl pathway and transcription factors. Table S6: cell cycle-related proteins modified by Ubls. Table S7: interactions of the Slx5-Slx8 complex in the MI network. Figure S1: clique degrees and sizes in the U-net and random networks. Figure S2: chromatin proteins regulated by Ub and SUMO. Figure S3: properties of ubiquitinated and sumoylated proteins. Figure S4: logo representation of the flanking regions of ubiquitinated lysines. File S1: plain text representation of the U-net. File S2: multiple sequence alignment of the SUS1 domain. [file gb-2009-10-3-r33-S1.zip › addFile1/index_files/image002.jpg]

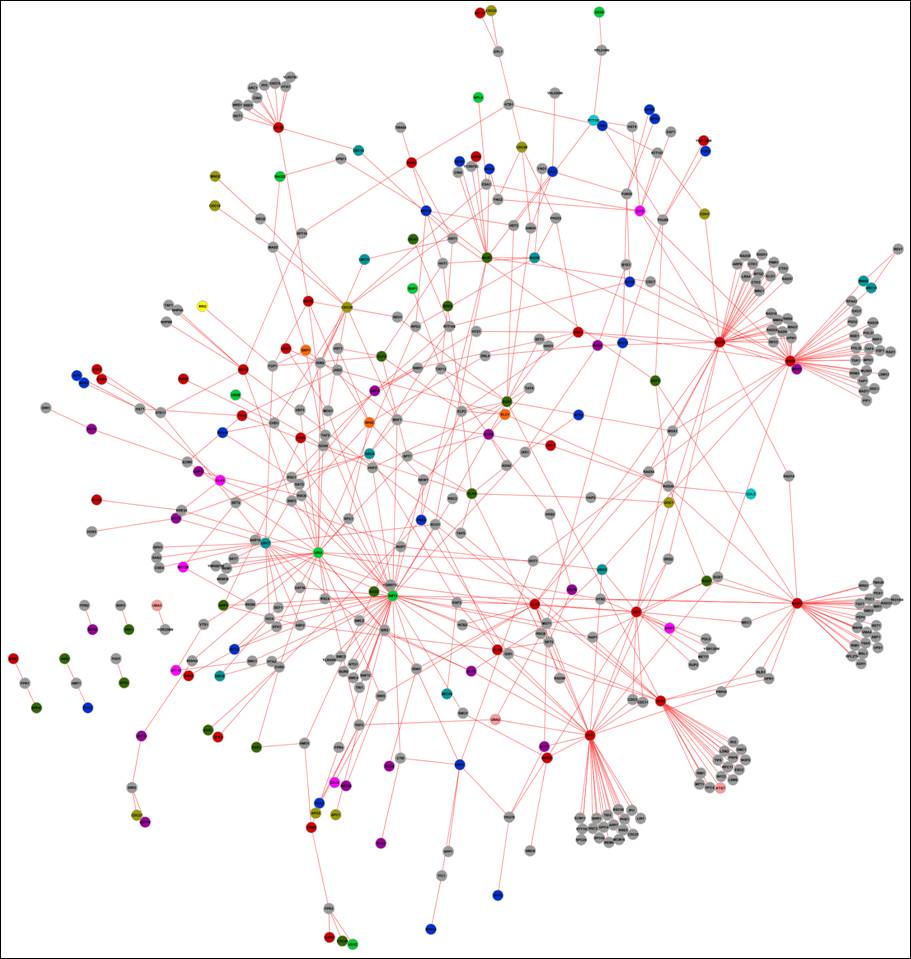

Supplement: Additional data file 1 — Table S1: annotations and additional information on all the U-net components. Table S2: modular structure of the U-net. Table S3: feedback regulation of the Ub/Ubl pathway. Table S4: ubiquitination/sumoylation and cellular localization. Table S5: Ub/Ubl pathway and transcription factors. Table S6: cell cycle-related proteins modified by Ubls. Table S7: interactions of the Slx5-Slx8 complex in the MI network. Figure S1: clique degrees and sizes in the U-net and random networks. Figure S2: chromatin proteins regulated by Ub and SUMO. Figure S3: properties of ubiquitinated and sumoylated proteins. Figure S4: logo representation of the flanking regions of ubiquitinated lysines. File S1: plain text representation of the U-net. File S2: multiple sequence alignment of the SUS1 domain. [file gb-2009-10-3-r33-S1.zip › addFile1/index_files/image006.jpg]

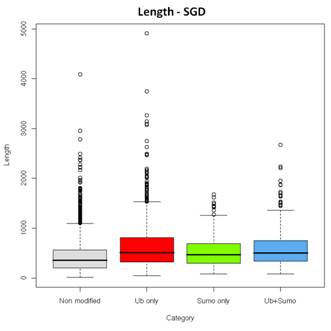

Supplement: Additional data file 1 — Table S1: annotations and additional information on all the U-net components. Table S2: modular structure of the U-net. Table S3: feedback regulation of the Ub/Ubl pathway. Table S4: ubiquitination/sumoylation and cellular localization. Table S5: Ub/Ubl pathway and transcription factors. Table S6: cell cycle-related proteins modified by Ubls. Table S7: interactions of the Slx5-Slx8 complex in the MI network. Figure S1: clique degrees and sizes in the U-net and random networks. Figure S2: chromatin proteins regulated by Ub and SUMO. Figure S3: properties of ubiquitinated and sumoylated proteins. Figure S4: logo representation of the flanking regions of ubiquitinated lysines. File S1: plain text representation of the U-net. File S2: multiple sequence alignment of the SUS1 domain. [file gb-2009-10-3-r33-S1.zip › addFile1/index_files/image025.jpg]

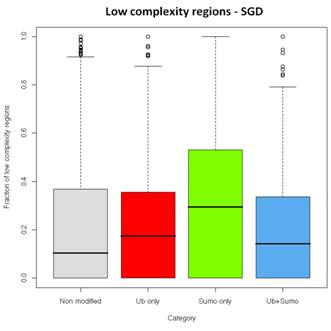

Supplement: Additional data file 1 — Table S1: annotations and additional information on all the U-net components. Table S2: modular structure of the U-net. Table S3: feedback regulation of the Ub/Ubl pathway. Table S4: ubiquitination/sumoylation and cellular localization. Table S5: Ub/Ubl pathway and transcription factors. Table S6: cell cycle-related proteins modified by Ubls. Table S7: interactions of the Slx5-Slx8 complex in the MI network. Figure S1: clique degrees and sizes in the U-net and random networks. Figure S2: chromatin proteins regulated by Ub and SUMO. Figure S3: properties of ubiquitinated and sumoylated proteins. Figure S4: logo representation of the flanking regions of ubiquitinated lysines. File S1: plain text representation of the U-net. File S2: multiple sequence alignment of the SUS1 domain. [file gb-2009-10-3-r33-S1.zip › addFile1/index_files/image026.jpg]

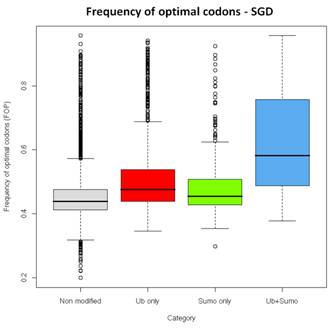

Supplement: Additional data file 1 — Table S1: annotations and additional information on all the U-net components. Table S2: modular structure of the U-net. Table S3: feedback regulation of the Ub/Ubl pathway. Table S4: ubiquitination/sumoylation and cellular localization. Table S5: Ub/Ubl pathway and transcription factors. Table S6: cell cycle-related proteins modified by Ubls. Table S7: interactions of the Slx5-Slx8 complex in the MI network. Figure S1: clique degrees and sizes in the U-net and random networks. Figure S2: chromatin proteins regulated by Ub and SUMO. Figure S3: properties of ubiquitinated and sumoylated proteins. Figure S4: logo representation of the flanking regions of ubiquitinated lysines. File S1: plain text representation of the U-net. File S2: multiple sequence alignment of the SUS1 domain. [file gb-2009-10-3-r33-S1.zip › addFile1/index_files/image027.jpg]

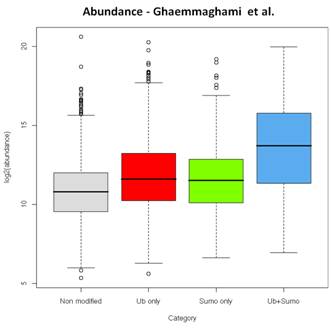

Supplement: Additional data file 1 — Table S1: annotations and additional information on all the U-net components. Table S2: modular structure of the U-net. Table S3: feedback regulation of the Ub/Ubl pathway. Table S4: ubiquitination/sumoylation and cellular localization. Table S5: Ub/Ubl pathway and transcription factors. Table S6: cell cycle-related proteins modified by Ubls. Table S7: interactions of the Slx5-Slx8 complex in the MI network. Figure S1: clique degrees and sizes in the U-net and random networks. Figure S2: chromatin proteins regulated by Ub and SUMO. Figure S3: properties of ubiquitinated and sumoylated proteins. Figure S4: logo representation of the flanking regions of ubiquitinated lysines. File S1: plain text representation of the U-net. File S2: multiple sequence alignment of the SUS1 domain. [file gb-2009-10-3-r33-S1.zip › addFile1/index_files/image028.jpg]

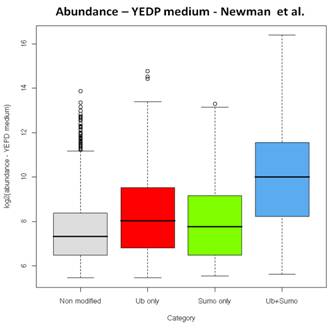

Supplement: Additional data file 1 — Table S1: annotations and additional information on all the U-net components. Table S2: modular structure of the U-net. Table S3: feedback regulation of the Ub/Ubl pathway. Table S4: ubiquitination/sumoylation and cellular localization. Table S5: Ub/Ubl pathway and transcription factors. Table S6: cell cycle-related proteins modified by Ubls. Table S7: interactions of the Slx5-Slx8 complex in the MI network. Figure S1: clique degrees and sizes in the U-net and random networks. Figure S2: chromatin proteins regulated by Ub and SUMO. Figure S3: properties of ubiquitinated and sumoylated proteins. Figure S4: logo representation of the flanking regions of ubiquitinated lysines. File S1: plain text representation of the U-net. File S2: multiple sequence alignment of the SUS1 domain. [file gb-2009-10-3-r33-S1.zip › addFile1/index_files/image029.jpg]

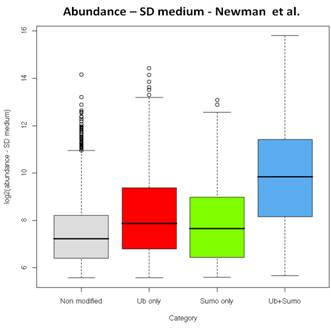

Supplement: Additional data file 1 — Table S1: annotations and additional information on all the U-net components. Table S2: modular structure of the U-net. Table S3: feedback regulation of the Ub/Ubl pathway. Table S4: ubiquitination/sumoylation and cellular localization. Table S5: Ub/Ubl pathway and transcription factors. Table S6: cell cycle-related proteins modified by Ubls. Table S7: interactions of the Slx5-Slx8 complex in the MI network. Figure S1: clique degrees and sizes in the U-net and random networks. Figure S2: chromatin proteins regulated by Ub and SUMO. Figure S3: properties of ubiquitinated and sumoylated proteins. Figure S4: logo representation of the flanking regions of ubiquitinated lysines. File S1: plain text representation of the U-net. File S2: multiple sequence alignment of the SUS1 domain. [file gb-2009-10-3-r33-S1.zip › addFile1/index_files/image030.jpg]

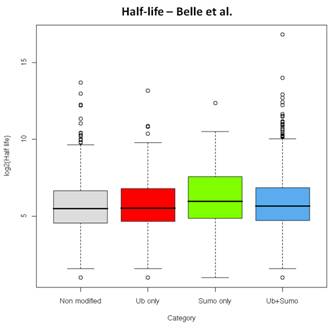

Supplement: Additional data file 1 — Table S1: annotations and additional information on all the U-net components. Table S2: modular structure of the U-net. Table S3: feedback regulation of the Ub/Ubl pathway. Table S4: ubiquitination/sumoylation and cellular localization. Table S5: Ub/Ubl pathway and transcription factors. Table S6: cell cycle-related proteins modified by Ubls. Table S7: interactions of the Slx5-Slx8 complex in the MI network. Figure S1: clique degrees and sizes in the U-net and random networks. Figure S2: chromatin proteins regulated by Ub and SUMO. Figure S3: properties of ubiquitinated and sumoylated proteins. Figure S4: logo representation of the flanking regions of ubiquitinated lysines. File S1: plain text representation of the U-net. File S2: multiple sequence alignment of the SUS1 domain. [file gb-2009-10-3-r33-S1.zip › addFile1/index_files/image031.jpg]

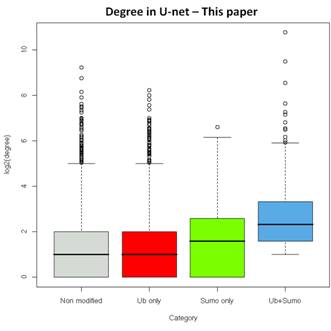

Supplement: Additional data file 1 — Table S1: annotations and additional information on all the U-net components. Table S2: modular structure of the U-net. Table S3: feedback regulation of the Ub/Ubl pathway. Table S4: ubiquitination/sumoylation and cellular localization. Table S5: Ub/Ubl pathway and transcription factors. Table S6: cell cycle-related proteins modified by Ubls. Table S7: interactions of the Slx5-Slx8 complex in the MI network. Figure S1: clique degrees and sizes in the U-net and random networks. Figure S2: chromatin proteins regulated by Ub and SUMO. Figure S3: properties of ubiquitinated and sumoylated proteins. Figure S4: logo representation of the flanking regions of ubiquitinated lysines. File S1: plain text representation of the U-net. File S2: multiple sequence alignment of the SUS1 domain. [file gb-2009-10-3-r33-S1.zip › addFile1/index_files/image032.jpg]

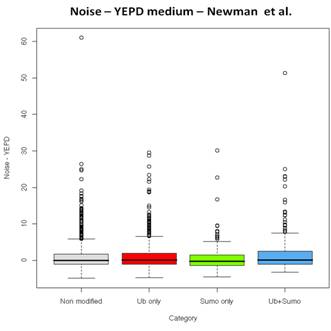

Supplement: Additional data file 1 — Table S1: annotations and additional information on all the U-net components. Table S2: modular structure of the U-net. Table S3: feedback regulation of the Ub/Ubl pathway. Table S4: ubiquitination/sumoylation and cellular localization. Table S5: Ub/Ubl pathway and transcription factors. Table S6: cell cycle-related proteins modified by Ubls. Table S7: interactions of the Slx5-Slx8 complex in the MI network. Figure S1: clique degrees and sizes in the U-net and random networks. Figure S2: chromatin proteins regulated by Ub and SUMO. Figure S3: properties of ubiquitinated and sumoylated proteins. Figure S4: logo representation of the flanking regions of ubiquitinated lysines. File S1: plain text representation of the U-net. File S2: multiple sequence alignment of the SUS1 domain. [file gb-2009-10-3-r33-S1.zip › addFile1/index_files/image033.jpg]

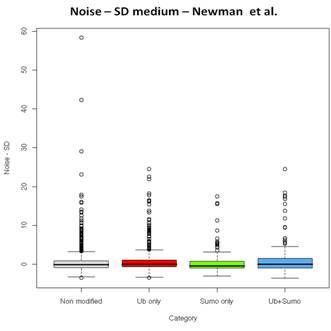

Supplement: Additional data file 1 — Table S1: annotations and additional information on all the U-net components. Table S2: modular structure of the U-net. Table S3: feedback regulation of the Ub/Ubl pathway. Table S4: ubiquitination/sumoylation and cellular localization. Table S5: Ub/Ubl pathway and transcription factors. Table S6: cell cycle-related proteins modified by Ubls. Table S7: interactions of the Slx5-Slx8 complex in the MI network. Figure S1: clique degrees and sizes in the U-net and random networks. Figure S2: chromatin proteins regulated by Ub and SUMO. Figure S3: properties of ubiquitinated and sumoylated proteins. Figure S4: logo representation of the flanking regions of ubiquitinated lysines. File S1: plain text representation of the U-net. File S2: multiple sequence alignment of the SUS1 domain. [file gb-2009-10-3-r33-S1.zip › addFile1/index_files/image035.jpg]

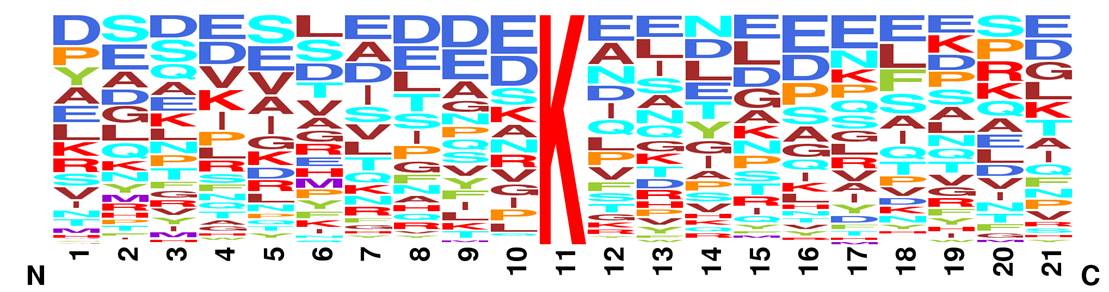

Supplement: Additional data file 1 — Table S1: annotations and additional information on all the U-net components. Table S2: modular structure of the U-net. Table S3: feedback regulation of the Ub/Ubl pathway. Table S4: ubiquitination/sumoylation and cellular localization. Table S5: Ub/Ubl pathway and transcription factors. Table S6: cell cycle-related proteins modified by Ubls. Table S7: interactions of the Slx5-Slx8 complex in the MI network. Figure S1: clique degrees and sizes in the U-net and random networks. Figure S2: chromatin proteins regulated by Ub and SUMO. Figure S3: properties of ubiquitinated and sumoylated proteins. Figure S4: logo representation of the flanking regions of ubiquitinated lysines. File S1: plain text representation of the U-net. File S2: multiple sequence alignment of the SUS1 domain. [file gb-2009-10-3-r33-S1.zip › addFile1/index_files/image038.jpg]
